# Effectiveness of a Cognitive Behavioral Therapy-Based Indicated Prevention Program for Children with Elevated Anxiety Levels: a Randomized Controlled Trial

**DOI:** 10.1007/s11121-016-0725-5

**Published:** 2016-11-08

**Authors:** Manon L. A. van Starrenburg, Rowella C. M. W. Kuijpers, Marloes Kleinjan, Giel J. M. Hutschemaekers, Rutger C. M. E. Engels

**Affiliations:** 1 Behavioural Science Institute, Radboud University Nijmegen, Montessorilaan 3, 6525 HR Nijmegen, The Netherlands; 2 Ambulatorium KJJ, Toernooiveld 5, Postbus 6909, 6503 GK Nijmegen, The Netherlands; 3 Pro Persona, Tarweweg 2, 6534 AM Nijmegen, The Netherlands; 4 Trimbos, Da Costakade 45, 3521 VS Utrecht, The Netherlands; 5 Utrecht University, Heidelberglaan 1, 3584 CS Utrecht, The Netherlands

**Keywords:** Anxiety, Children, Prevention, CBT, Group intervention

## Abstract

Childhood anxiety is a problem not only because of its negative consequences on the well-being of children but also because of its adverse effects on society and its role in mental disorders later in life. Adequate prevention might be the key in tackling this problem. The effectiveness of Coping Cat, as an indicated CBT-based prevention program in Dutch primary school children, was assessed by means of a randomized controlled trial. In total, 141 children aged 7–13 with elevated levels of anxiety and their mothers were included and randomly assigned to an intervention group and a waiting list control group. After screening, all participants completed baseline, post-intervention, and 3-month follow-up assessments. The results showed that Coping Cat, as an indicated prevention program, reduces children’s self-reported anxiety symptoms, with Cohen’s effect size *d* of 0.66 at the 3-month follow-up. A moderating effect was found for baseline anxiety level; specifically, children with high levels of baseline anxiety who received the Coping Cat program had lower anxiety levels at follow-up compared to children with high levels of anxiety in the control condition. No moderating effects of gender or age were found. An unexpected decline in anxiety levels from screening to pre-assessment was found in both groups, and this decline was stronger in the experimental group. These promising results warrant the implementation of Coping Cat as an indicated prevention program.

## Introduction

Anxiety disorders are among the most prevalent mental disorders among children and adolescents (Kroes et al. 2001; Tuebert and Pinquart 2011; Verhulst et al. 1997). About 75% of anxiety disorders have an onset between 11 and 21 years of age (Kessler et al. 2005). Anxiety disorders can have detrimental consequences, both short and long term, on emotional and social functioning of children (Regier et al. 1998). Nevertheless, the vast majority of children and adolescents with high levels of anxiety do not get the necessary treatment (Essau 2003), resulting in a substantial number of children and adolescents with unnoticed and untreated subclinical and clinical anxiety. When childhood anxiety disorders are left untreated, they are known to persist into adulthood (Reef et al. 2009), which not only prolongs the affected individuals’ suffering but also increases health care costs (Bodden et al. 2008). The early onset of anxiety disorders and associated negative effects as well as the high number of youths not receiving the treatment, highlights the need for early screening and prevention.

According to several meta-analyses, anxiety prevention in youth has shown to be effective (Mychailyszyn et al. 2012; Tuebert and Pinquart 2011). This overview offers clear recommendations on how to increase the effectiveness of an anxiety prevention program. First, selective and indicated anxiety prevention are proven to be more effective compared to universal anxiety prevention (Tuebert and Pinquart 2011). Second, the effectiveness of anxiety prevention increases when the administered program focuses directly on anxiety symptoms instead of broader symptoms characterizing also other disorders, e.g., both anxiety and depression. Third, anxiety prevention works best when provided by a mental health professional. The fourth important topic regarding the implementation of prevention programs is its cost-effectiveness. Screening and offering a child-focused intervention to children with elevated levels of anxiety was found to be more cost-effective compared to “doing nothing” and waiting, which implies waiting until symptoms grow into a full-blown disorder that requires mental health treatment (Simon et al. 2013). Although the meta-analysis of Tuebert and Pinquart (2011) offered clear guidelines on how to increase the effectiveness of anxiety prevention, only few studies in their extensive review met most of these recommendations. First, the most widely used anxiety prevention programs (e.g., the FRIENDS program or Penn Resilience Program) often lack a sole focus on anxiety. Second, exposure techniques are used scarcely, despite their central role in decreasing anxiety (Olatunji et al. 2009). Finally, programs are not freely available, which hinders the cost-effectiveness and dissemination. Clearly, this demonstrates the need for an indicated prevention program that would incorporate all recommendations for effective anxiety prevention. Instead of developing a new program to meet these requirements, a more effective and time-saving approach is to use a program that has been proven to be effective in a clinical sample and meets the requirements of prevention mentioned earlier.

The cognitive behavioral therapy (CBT)-based Coping Cat program has potential to meet all requirements of prevention. The Coping Cat program focuses on anxiety-specific symptoms and emphasizes exposure. The group format is attractive for school-based use, and the program is freely accessible to therapists. In addition, group-based programs are likely to be more cost-effective compared to individual programs. In youth, Coping Cat has already been proven effective as an intervention treatment program for children with clinical anxiety in several studies conducted both in the USA (Kendall et al. 1997, 2008) and in other countries, including The Netherlands (Barrett et al. 1996; Nauta et al. 2003). Although most of these effectiveness studies focused on treating individuals, a few studies that focused on the group intervention have also proven its effectiveness in a clinical sample (Flannery-Schroeder and Kendall 2000), and they have shown that it could be effective in reducing anxiety levels in a clinical setting, even at the 1-year follow-up (Flannery-Schroeder et al. 2005). However, this evidence-based group program has never been tested in a school-based setting in a population of children with elevated levels of anxiety; thus, its effectiveness as an indicated prevention group program has yet to be determined.

To conclude, the first aim of this study was to evaluate the effectiveness of a Dutch version of Coping Cat as a group-based indicated prevention program in a randomized controlled trial (RCT) (van Starrenburg et al. 2013). In addition, age, gender, and baseline anxiety severity scores were examined as moderators of the treatment, since these are known to be predictors of the effectiveness of an anxiety treatment (Bennett et al. 2013).

## Method

### Sample Selection

Five primary schools in The Netherlands distributed an information letter to the parents of all children in grades 3 through 6. To assure a representative sample of children with elevated anxiety levels was selected, we used passive parental consent for screening. Active parental consent was required for children to participate in the intervention and the study. In The Netherlands, this procedure is often approved because screening is not necessarily considered a part of the actual study. The ethics committee of the Faculty of Social Sciences at the Radboud University Nijmegen (ECG2012-0910-053) approved this study. The trial is registered at the Dutch Trial Register (NTR3818).

A total of 639 (94% response rate) children were screened (T0) using the Dutch version of the Spence Children’s Anxiety Scale (SCAS) (Muris et al. 2000). The SCAS was developed to assess anxiety symptoms in children, and it has proven to be a reliable and valid instrument in The Netherlands (Muris et al. 2000; Nauta et al. 2004). To select children with elevated (above average) anxiety levels, we used 1 SD above our sample mean as a cut-off instead of using the clinical cut-off of the SCAS. Children with an anxiety level of 1 SD above the sample mean on the total or one of the subscales were identified as eligible. All cut-offs were calculated for boys and girls separately. We notified all parents about their child’s anxiety levels, contacted the parents of children with elevated anxiety levels by telephone, and informed them about the potential participation of their child in the anxiety program. All parents provided active parental consent for the children’s participation in the study from this point forward. Due to a mistake made in SPSS during screening, 13 girls with an elevated social phobia score who could have been included in the RCT were mistakenly missed and not approached to participate in the study. Furthermore, children with an elevated score based *solely* on the obsessive-compulsive disorder scale were excluded from the study, since studies show that they benefit more from a specific treatment (Barrett et al. 2008); thus, the present program could not target this anxiety properly.

High anxiety levels were not considered a reason for exclusion, since CBT is also the first-choice treatment for clinical anxiety. However, we included one item from the Child Depression Inventory (CDI) to assess suicide at pre-assessment, post-assessment, and follow-up to detect children in severe distress, as anxiety is known to be comorbid with depression. A therapist contacted the parents of four children (one child at pre- and three children at post-assessment) who scored high on this item. Only one child (post-assessment) was referred to mental health care. None of the children needed to be excluded from the study. Finally, ten children who were receiving CBT at the time of the study, or who had received it in the past year, were excluded. Overall, 141 eligible children and their mothers agreed to participate.

**Fig. 1:**
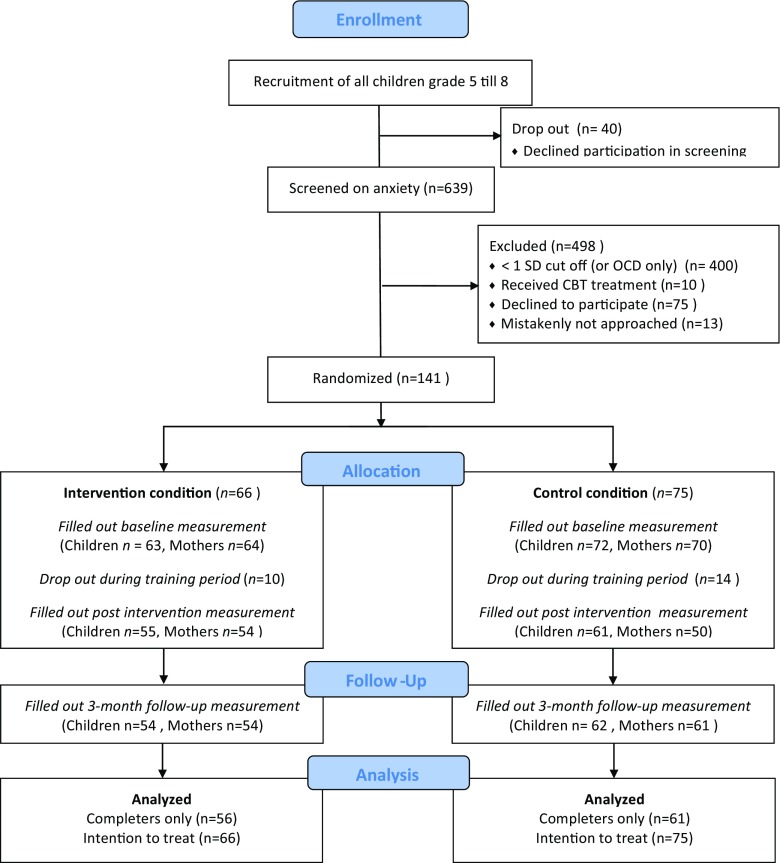
Flowchart of recruitment, randomization, follow-up, and analyses

Randomization was conducted within schools to control for school characteristics. Children were stratified by age (young 7–9 years and old 10–12 years) and assigned to both conditions proportionally. An independent researcher from the research institute used a computerized random number generator with a blocked randomization scheme to perform the allocation. All schools had an equal number of experimental and control groups, with the exception of one school comprising one experimental and two control groups, resulting in 66 children in the intervention condition and 75 children in the control condition. To prevent an expectancy effect, we informed the children and their mothers about the assigned condition *after* completing the baseline measurement. Further information about the participant flow from screening through follow-up is included in Fig. 1.

### Participants

Overall, 141 children and their mothers participated in this study. Children in third through sixth grades of elementary school were included (mean age = 9.48 years, SD = 1.27). Slightly more girls participated (55.3%). Overall, 76% of children lived in a two-parent household with both their biological parents. The mothers had a mean age of 41.14 years, SD = 4.07. Most mothers (55%) finished a vocational education, and about 25% had college or higher education. Approximately 40% of the families had a low to average income. The majority of children (92.9%) and mothers (90.8%) were born in The Netherlands.

### Procedure

All participating children and their mothers (experimental and control group) completed baseline measurements (T1) 2 to 3 weeks before the training started. A post-assessment (T2) was done 2 to 3 weeks after the completion of the training. The 3-month follow-up (T3) was conducted 3 months after finishing the training. At T1–T3, all children completed the suicidal ideation item of the CDI to check whether immediate care was needed. Maternal data was collected at T1. Children in the control condition were given the opportunity to participate in the Coping Cat program after the 3-month follow-up assessment. Weekly measurements were done in both the experimental and the control group to identify potential mediators. These last findings will be published in a separate paper.

### Program Prevention

To use the US group version of Coping Cat for prevention purposes (Flannery-Schroeder and Kendall 1996), we adapted the duration of the program (reducing the number of sessions from 18 to 12) and decreased contact time (from 2 to 1 h). The purpose of these adaptations was to better align the prevention program to a school-based context and to make it more cost-effective. To be consistent in translation and attend to potential cultural differences, the translation and wording were identical to the already existing Dutch version of the individual-oriented program for a clinical population (Nauta and Scholing 1998), which was found to be effective in the Dutch population (Nauta et al. 2003). The program can be obtained from the first author upon request.

#### Experimental Condition

Children in the experimental condition received 12 weekly 1-h sessions in groups of seven to nine children. The program took place in schools after regular school hours. During the first five sessions of the program, a rationale for anxiety was provided and several supportive techniques were taught (i.e., relaxation, challenging thoughts, and problem-solving). In session, exposure was introduced, starting with low-anxiety-provoking situations that the children had to confront as a group, which was followed by high-anxiety-provoking situations that they had to confront individually. During the program, parents received written information about the content of the program and the progress of their child. For a detailed description of each session, we refer to the study protocol (van Starrenburg et al. 2013).

#### Control Condition

Children in the control condition received no intervention and only filled out the questionnaires.

#### Trainers

Trainers (*N* = 5) were child psychologists with considerable experience in youth mental health care and CBT. All trainers participated in a 2-day training followed by a 2-h supervision session conducted three times over the course of the program, to maintain treatment integrity. A master’s student in clinical child psychology assisted each therapist. One of the therapists is a co-author of this paper.

### Measures

#### Anxiety

The primary outcome measure was the children’s anxiety level measured using the Dutch version of the Spence Children’s Anxiety Scale (SCAS). The SCAS comprises six subscales assessing panic attack and agoraphobia, separation anxiety disorder, social phobia, physical injury fears, obsessive-compulsive disorder, and generalized anxiety disorder. The child version comprises 44 items, with six positively worded filler items measured on a four-point scale. The parent-report (P) version comprises 38 items measured on the same four-point scale.

We used the mean SCAS score, referred to as “SCAS total,” to detect changes in overall anxiety levels. Cronbach’s alpha of this scale ranged from .88 to .91 across time points (T0–T3), which is consistent with prior studies (Muris et al. 2000). The alphas for the mother-reported overall child anxiety level named “SCAS-P total” ranged from .84 to .90 across time points (T0–T3), which is also consistent with prior studies (Nauta et al. 2004).

### Strategy of Analysis

#### Power

Research on indicated anxiety prevention (Tuebert and Pinquart 2011) has shown that small effect sizes (Hedges *g* .19) can be expected at the 1-month to 12-month follow-ups. Sample size calculations indicated that 65 participants needed to be included in each condition with the 3-month follow-up SCAS score as the main outcome. We expected that about 30% of the screened children would have elevated levels of anxiety. Out of all children with elevated levels of anxiety, we expected about 60% to participate in further study; therefore, about 680 children needed to be approached.

#### Attrition

We conducted logistic regression analyses to analyze attrition at T0 trough T1, with enrollment (“subjects who enrolled versus declined to enroll”) as the dependent variable and anxiety levels at screening, gender, and age as predictors. Participants with elevated levels of anxiety who declined to participate in the study were more likely to have lower levels of anxiety at screening (OR = 3.59, CI 95% = 1.51–8.56, *p* < 0.01). The results indicated no differences for gender or age group.

#### Analyses

To test for baseline differences between the two conditions, independent *t* tests and chi-square analysis were used. In accordance with the intent-to-treat principle, all children randomized to a condition were included in the analyses to test the study hypotheses (intention-to-treat (ITT) *N* = 141). The completers only group was also analyzed (*N* = 117). In the ITT group, the missing values on the primary outcome variable (anxiety level SCAS/SCAS-P) were imputed for all four measurements using 20 imputation sets by means of multiple imputations in SPSS 19. Imputations were done separately for the control group and experimental group. Variables that correlated significantly with the children’s anxiety levels were used as auxiliary variables (Graham 2009).

The 3-month follow-up measurement of children’s anxiety levels was the main outcome. Regression analyses tested whether children in the experimental condition showed a stronger decrease in anxiety symptoms at the 3-month follow-up compared to the control condition. Since randomization took place within the school level and children were “nested” within these schools, we used Mplus 6.1 (Muthen and Muthen 1998) to control for potential clustering effects. Baseline anxiety levels and variables that differed across conditions at baseline were included as covariates. The effect sizes and confidence intervals indicated both the magnitude and the effect of the prevention program. Moderating effects of gender, age, and baseline anxiety were tested by computing the interaction effects of these variables with condition. The standardized regression coefficients will be reported.

## Results

### Descriptive Statistics

In the total sample of 639 children, 239 children scored 1 SD above the mean on anxiety on one or more subscales. Most children scored high on one subscale (16.1%). See Table 1 for details. No differences were found between the control group and the experimental group in gender (*χ* ^2^ [1] = 0.726, *p* = 0.39), class level (*χ* ^2^ [3] = 4.69, *p* = 0.20), and social economic status (*χ* ^2^ [6] = 2.556, *p* = 0.86). Table 2 shows the means, standard deviations, and *t* values for the SCAS/SCAS-P “total” scores at T0–T3 for the experimental and control groups. The children in the control group obtained significantly higher scores on anxiety symptoms compared to the children in the experimental group at T1, T2, and T3. At T0, the experimental and control groups did not differ significantly in anxiety symptoms. Similar results were found for the ITT group. For the mother reports, no significant differences emerged between groups in children’s anxiety levels at any time point.

**Table 1:** Number of children with elevated levels of anxiety, per subscale and per number of subscale at screening

Amount of elevated subscales	Number of children	Percent	Scale SCAS	Number of children with elevated levels
0	400	62.6	Separation anxiety	74
1	103	16.1	Social anxiety	117
2	40	6.3	Panic	89
3	29	4.5	Phobia	112
4	28	4.4	Generalized	105
5	25	3.9	Total scale	94
6	14	2.2		
Total	639	100

**Table 2:** Means, standard deviations, and *t* values for the SCAS/SCAS-P “total” scores at T0, T1, T2, and T3 for the experimental group and control group (completers only sample)

	SCAS			SCAS-P		
	Experimental M (SD)	Control M (SD)	*t* value (*df*)	Experimental M (SD)	Control M (SD)	*t* value (*df*)
T0 (scr)	0.90 (.37)	0.97 (.33)	1.22 (139)	–	–	–
T1 (pre)	0.75 (.34)	0.91 (.36)	2.60 (133)*	0.51 (.21)	0.53 (.25)	0.47 (132)
T2 (post)	0.62 (.32)	0.85 (.45)	3.20 (114)**	0.43 (.16)	0.49 (.26)	1.48 (107)
T3 (FU)	0.53 (.35)	0.77 (.39)	3.43 (114)**	0.39 (.21)	0.46 (.28)	1.33 (113)

*p < 0.05, **p < 0.01

### Program Effectiveness

To evaluate the program’s effectiveness, outcome variables included child anxiety levels, as reported by the children themselves as well as by their mothers, at T2 and T3. The data for children and mothers are described separately, and the results for both groups are reported.

#### Child Report Data

**Fig 2:**
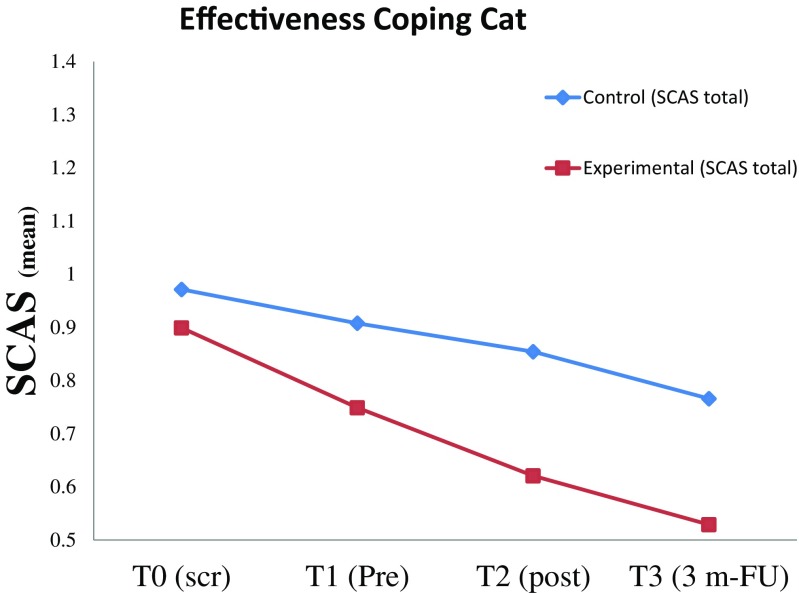
SCAS “total” scores (ITT group) at various time points

Across both groups, children’s self-reported anxiety levels declined over time (see Fig. 2). Between T0 and T3, the experimental group showed greater decrease in anxiety levels compared to the control group (SCAS total; *ß* = −0.283, *p* < 0.001). The results indicated a medium to large effect size for the children’s anxiety level at T3 (SCAS total; Cohen’s *d* = −0.66) and a small effect size T3 when adjusting for the significant difference in anxiety level between the two groups at T1 (SCAS total; Cohen’s *d* = −0.48). Between T0 and T1, the reported anxiety levels declined significantly for both groups, although it was stronger for the experimental group compared to the control group (ITT (SCAS total) *ß* = −0.164, SE = 0.05, *p* = 0.000). These findings were similar for both the ITT group and the completers only (CO) group. See Table 3.

**Table 3:** Linear regression models of the relation between anxiety level (SCAS total) and time

Time points	Child report data		Mother report data	
	ITT	CO	ITT	CO
	SCAS total *ß* (SE)	SCAS total *ß* (SE)	SCAS-P total *ß* (SE)	SCAS-P total *ß* (SE)
T0–T3	−0.28** (0.06)	−0.26** (0.04)	–	–
T1–T3	−0.21* (0.07)	−0.17** (0.07)	−0.12 (0.06)	−0.07** (0.03)

*ITT* intention-to-treat sample, *CO* completers only sample, *SE* standard error

*p < 0.05, **p < 0.01

At pre-assessment, 67% of the experimental group and 85% of the control group reported elevated levels of anxiety. At the 3-month follow-up, 38% of the experimental group compared to 59% of the control group reported elevated levels of anxiety. This means that after the program, almost two thirds of the experimental group experienced normal anxiety levels while almost two thirds of the control group still reported elevated levels of anxiety.

#### Mother Report Data

The children’s anxiety scores on the SCAS-P total scale, as reported by mothers, showed a significant effect only in the CO group between T1 and T3 (Table 3).

### Moderation

For the 3-month follow-up outcomes, no moderation effects were found for either age or gender. This was the case for both the ITT and CO child-reported and mother-reported data. We found a moderating effect of the child’s anxiety level at T0. Children with high levels of baseline anxiety who also received the Coping Cat program had lower anxiety levels (SCAS total) at follow-up compared to children with high levels of anxiety in the control condition. These effects were similar for both ITT (OR = 0.285, CI 95% = 0.164–0.406, *p* = 0.018) and CO (OR = 0.261, CI 95% = 0.184–0.338, *p* = 0.001). See Table 4 for all these results.

**Table 4:** Moderated regression analysis

	Child report data		Mother report data	
	ITT	CO	ITT	CO
	SCAS total *ß* (SE)	SCAS total *ß* (SE)	SCAS-P total *ß* (SE)	SCAS-P total *ß* (SE)
Gender	−0.32 (0.26)	−0.45 (0.30)	−0.14 (0.23)	−0.18 (0.12)
Anxiety level	0.29* (0.12)	0.26** (0.08)	−0.18 (0.28)	−0.21 (0.12)
Age	0.04 (0.54)	0.03 (0.26)	0.07 (0.57)	0.50 (0.63)

*ITT* intention-to-treat sample, *CO* completers only sample, *SE* standard error

*p < 0.05, **p < 0.01

## Discussion

The aim of the present study was to evaluate the effectiveness of Coping Cat as an indicated prevention program in Dutch primary school children with elevated levels of anxiety. The results showed that from the start of the training to the 3-month follow-up assessment, children’s self-reported anxiety levels decreased significantly more in the experimental group compared to the control group. The same pattern was found for the maternal reports of child anxiety (SCAS-P), although significant result emerged only in the CO group for the total SCAS scale. Furthermore, after the program, almost two thirds of the experimental group returned to anxiety levels that fell into the normal range while almost two thirds of the control group still reported elevated levels of anxiety. These findings indicated that the Coping Cat group program reduced elevated levels of anxiety among primary school children and that these effects remained 3 months after the training.

An unexpected decline in anxiety levels from screening to pre-assessment was found in both groups, although this effect was stronger in the experimental compared to the control group. These results cannot be attributed to selection bias or other treatment effects, as children who were in active treatment were excluded from participation and a strict randomization process was followed. The influence of the participants’ knowledge of their allocation to the experimental or the control group between the screening and pre-assessment, a so-called expectancy effect (Arrindell 2001; Wijnhoven et al. 2013), can also be ruled out because the participants were unaware of their allocation until after pre-assessment. A “test-retest effect,” wherein a change in mean scores towards less psychopathology between two measurements are observed without any formal intervention having yet taken place, could explain a decline in outcome measures between the two measurements. Multiple explanations can be offered to account for this effect, including mood-congruent associative processing, natural coping mechanisms, self-monitoring hypothesis, and response shift (Arrindell 2001). Simon et al. (2011) stated that the attention given to the child’s anxiety during assessments could explain this phenomenon in that increased attention could increase children’s openness to and awareness of anxiety and its consequences, resulting in reduced anxiety. However, none of these reasons explains the larger decrease in anxiety among the participants in the experimental group. At this moment, we have no other explanation for this finding other than the result is due to chance or unknown factors. However, because a test-retest effect between two measurements has been frequently reported in the literature, it is remarkable that only few studies, e.g., Scholten et al. 2016, reported using two measurement points before the formal intervention to make sure that a potential effect can be attributed to a real intervention effect instead of a test-retest effect. In our study, we found a real intervention effect, and we would like to encourage other scholars to also use two measurement points before starting the actual intervention.

This study has several limitations. First, a low-attentive control group (measurements only) was used while research (Neil and Christensen 2009) suggests using a more active control condition. To distinguish between intervention effects and effects due to other factors, an active control group that would not be subjected to CBT techniques should be used. Factors such as social support and attention should also be considered. Although our control group received treatment as usual and weekly adult attention when completing the questionnaires, comparing Coping Cat to an active control group could give a more precise conception of the unique effect of CBT and decrease the influence of biases. Second, since the children completed the questionnaires about their anxiety weekly, a measurement-induced improvement could occur (Knowles et al. 1996). Although these weekly measurements can give us valuable insights on how the program works, anxiety levels decreased in both groups partly due to a repeated measurements effect. However, a stronger decrease in the experimental group at T3 suggests an additional effect of the prevention program. Third, because of compatibility with indicated prevention purposes, the selection of the participants with elevated levels of anxiety was purposely done using an anxiety questionnaire rather than a (semi)-structured diagnostic interview. One of the main disadvantages of using a questionnaire for screening purposes is that it does not allow for objective professional assessment of the anxiety levels, which might decrease inclusion reliability. It would be too burdensome to use interviews to screen for children with only elevated levels of anxiety, and it would also lower the cost-effectiveness. One solution could be to use gate items or administer the screening twice to exclude false positives (Lucas et al. 2001). Fourth, this trial was not set up to compare smaller subgroups based on the type of anxiety; thus, potential differences among these subgroups in their response to the intervention are difficult to determine. Further research should include a larger sample, consider the type of anxiety in the power calculation and randomization process, and use other statistical approaches, such as cluster of mixture analysis. Finally, only short-term effects of Coping Cat (3-month follow-up) were obtained. It is debatable whether 3 months is sufficient to demonstrate the full effect of the intervention. However, several meta-analyses (Fisak et al. 2011) showed that effect sizes at the 6- and 12-month follow-ups were comparable to those at post-intervention, suggesting that the response to the prevention programs is maintained at longer follow-up periods. Studies with follow-ups longer than 12 months are scarce, although (Simon et al. 2011) they reported similar results. More research is needed to provide better insights into the long-term effectiveness and effect of the Coping Cat group program on children’s functioning and vulnerability to future anxiety problems.

Several implications for clinical practice emanate from this study. The findings of this study suggest that implementation of the Coping Cat as an indicated prevention program in schools might be considered in the future. The current study was set up to closely resemble a “real life” setting, which enhances the generalizability of the results. Because of its group-based setup, the program also facilitates the implementation process and increases the cost-effectiveness. In the current study, trained psychologist facilitated the program; however, the cost-effectiveness may improve if school counselors were trained to deliver the program. Accordingly, schools would not have to hire external and expensive psychologists. Further research is necessary to explore this possibility, as the existing studies show contradicting results (O’Leary-Barrett et al. 2010). Another consideration is the accessibility of the program. We suggest a free availability of the booklet itself for schools and trainers, which would decrease the costs and facilitate easy use of the program within the educational system. To substantiate these recommendations, further research on the cost-effectiveness, implementation, and long-term effects of the Coping Cat prevention program is recommended.

In summary, the Coping Cat group program has been found effective in reducing child-reported anxiety levels in primary school children with elevated levels of anxiety at the 3-month follow-up. Even though several ways can be used to control for potential placebo effects and increase the intervention effects even further, the current results supported the effect of the program in the intervention compared to a low-attentive control group. Moreover, this program is group-based, school-based, and freely accessible; hence, it already meets several cost-effectiveness criteria and shows promise for the implementation.
